# The use of functional amino acids in different categories of pigs – A review

**DOI:** 10.17221/72/2023-VETMED

**Published:** 2023-08-27

**Authors:** Nikola Hodkovicova, Simon Halas, Kristina Tosnerova, Kamil Stastny, Martin Svoboda

**Affiliations:** ^1^Department of Infectious Diseases and Preventive Medicine, Veterinary Research Institute, Brno, Czech Republic; ^2^Department of Animal Nutrition and Husbandry, University of Veterinary Medicine and Pharmacy in Kosice, Kosice, Slovak Republic; ^3^Ruminant and Swine Clinic, University of Veterinary Sciences Brno, Brno, Czech Republic

**Keywords:** epithelial barrier, oxidative stress, oxidative stress, pregnancy, sow, weaning

## Abstract

The present review deals with a particularly important topic: the use of functional amino acids in different categories of pigs. It is especially relevant in the context of the current efforts to reduce the use of antibiotics in pig farming and the search for possible alternatives to replace them. The review is based on the definition that functional amino acids (FAAs) are classified as dispensable amino acids, but with additional biological functions, i.e., not only are they used for protein formation, but they are also involved in regulating essential metabolic pathways to improve health, survival, growth, and development. We describe the mechanism of action of individual FAAs and their potential use in pigs, including glutamate, glutamine, arginine, branched-chain amino acids (i.e., leucine, isoleucine, and valine), tryptophan and glycine. The work is divided into three parts. The first part deals with the FAAs and their role in the overall health of sows and their offspring. The second part describes the use of functional amino acids in piglets after weaning. Part three examines the use of functional amino acids in growing and fattening pigs and their impact on meat quality.

## INTRODUCTION

The present review deals with the use of functional amino acids (FAAs) in different categories of pigs. The substitution with FAAs is considered an important topic due to several reasons that mainly include the proper nutritional and physiological status of pigs, lowering of antibiotic resistance, and economic factors (Kim et al. 2007). Their main role in the organism is their use for the synthesis of proteins and other necessary substances; their oxidation acts as a source of energy, and they regulate the key metabolic pathways including oxidative stress, immunity, and intestinal barrier protection (Wu 2013; Le Floc’h et al. 2018; Prates et al. 2021).

In the last decade, the focus in the area of pig production has been mainly on the introduction of hyper-prolific sows because an increase in the number of live-born piglets and weaned piglets per cycle means a greater economic return for producers. Optimising nutrition is generally accepted as a very important prerequisite for meeting these higher performance standards while maintaining the health and welfare of sows. An effective and comprehensive animal health programme includes many components, one of the essential factors being adequate nutrition and the use of feed additives. One of the ways to contribute to the solution of these problems is the use of FAAs (Kim et al. 2007; Prates et al. 2021).

Next, there is an issue with antibiotic resistance discussed commonly in the last decades, and alternative approaches are sought (Beaumont et al. 2022). The effort to limit resistance to antibiotics has led to the creation of new rules for their use in diet and water for farm animals; nowadays, it is possible to administer antibiotics only therapeutically and not for prophylactic purposes [Regulation (EU) 2019/6 of the European Parliament and of the Council]. Since 2006, a complete ban on the use of antibiotic growth promotors has also been in force in the European Union states. The aim is to reduce the use of antibiotics and, when feasible, replace them with alternative procedures. Additionally, zinc oxide was used for many years to reduce diarrhoea in piglets after weaning; however, the EU regulation from June 2022 banned the use of zinc oxide for therapeutic use because of its potential risk to increase environmental pollution and a potential to increase the resistance of certain bacterial species (Pejsak et al. 2023). In both, antibiotics and zinc oxide, the use of FAAs can be a suitable alternative. In general, amino acids are categorised into three groups: essential, semi-essential (conditionally essential), and non-essential amino acids, depending on their dietary essentiality and role in protein synthesis (Mou et al. 2019). FAAs are customarily defined as those amino acids that are not only used to build proteins but that participate in extra biological functions, such as regulating essential metabolic pathways to improve health, survival, growth, and development (Kim et al. 2007; Ji et al. 2020). The role of FAAs in the organism was summarised as follows: 1) provide substrates for the synthesis of tissue protein and regulate their degradation, 2) impact hormone synthesis and secretion, 3) regulate endothelial function, vasodilation, and blood flow, 4) affect nutrient metabolism, and maintain acid-base balance and whole-body homeostasis (Kim et al. 2007; Wu 2009; Wu 2013).

Amino acids and fatty acids with special functions include arginine, branched-chain amino acids (BCAA; i.e., leucine, isoleucine, and valine), glutamate, glutamine, tryptophan, glycine, and taurine (Kim et al. 2007). Leucine, isoleucine and valine are substrates for glutamine synthesis in animal tissues and, therefore, glutamine may partly mediate the anabolic effect of the branched-chain amino acids in animals (Wu 2009). The well-investigated functional amino acids are the arginine family, which includes arginine, glutamine, glutamate, aspartate, proline, etc. (Ji et al. 2020).

## THE FAAS AND THEIR ROLE IN THE OVERALL HEALTH OF SOWS AND THEIR OFFSPRINGS (SUCKLING PIGLETS)

Modern genetic lines of sows have an average of 14 to 16 functional teats that can provide adequate nutrition for suckling piglets. A sow that gives birth to more piglets than is her number of functional teats is classified as a hyperprolific sow (Oliviero 2022). All over the world, there are special genetic programs that manage crossbreeding with the aim of finding the best possible ratio of the quantity of newly born individuals and the number of litters per year to the quality and survival of the litters, growth and carcass traits (Moeller et al. 2004). In Europe, the commercial females used are mainly crosses of the breed Large White, Landrace and Yorkshire; for example, the DanBred is the most preferred hybrid across Europe originated from Denmark and was developed by crossing the Yorkshire and Landrace bred and subsequently their offspring (F1 generation) with Duroc (Danbred 2023). In general, the modern hyperprolific sows currently have litters with an average number of piglets of 18–20 individuals, which leads to an increase in mortality both after farrowing and before weaning, to reduced birth weight of piglets in general, but also to an extended time of farrowing (Oliviero 2022).

Enhancement of foetal growth is important to avoid early embryonic death of the foetus (mainly from day 12 to day 25 after insemination), and then during pregnancy (mainly from day 35 to day 75), when insufficient development of the placenta or insufficiency of uterine capacity may occur (Blavi et al. 2021). This can result in an inadequate supply of oxygen and nutrients to the foetus. Dietary supplements can be used to support the growth and proper functioning of the placenta; these mainly include sufficient amounts of nutrients that can simultaneously increase the number of piglets in the litter and support their growth, for example, chromium, L-carnitine, L-arginine, omega fatty acids, lysine and FAAs (Ji et al. 2020; Blavi et al. 2021). It was found that piglets born to sows fed increased amounts of FAAs in their diet during pregnancy are more viable after birth, have increased colostrum intake and have higher weight gains (Nuntapaitoon et al. 2018).

### L-arginine

One of the most required FAAs for pregnant sows and newborn piglets, L-arginine, stimulates the secretion of insulin, growth hormone, prolactin, glucagon, and placental lactogen (Blachier et al. 2013). Administration of higher doses of L-arginine during early pregnancy has been observed to have a positive effect on the development of the placenta, as well as supporting lactation, increasing fertility, and promoting foetal growth (Mou et al. 2019). In general, L-arginine undergoes metabolic processing in the body, which subsequently produces nitric oxide, creatinine, and polyamines (Nuntapaitoon et al. 2018). All these substances are necessary at the beginning of pregnancy for the proper development and growth of the foetus, angiogenesis and the development of the placenta (Ji et al. 2020). However, the duration of supplementation and the most appropriate time to administer L-arginine are still the subject of many studies. For instance, its positive effect on the number of live-born piglets was observed when 0.5% or 1% L-arginine was administered to pregnant sows from day 85 of pregnancy until farrowing. When pregnant sows were supplemented with 0.5% L-arginine from day 85 of pregnancy to the day of parturition, there was also an increase in oxygen saturation and an increase in the body weight of newborn piglets (Nuntapaitoon et al. 2018).

L-arginine was found to be one of the most important FAAs for suckling piglets as well. The beneficial effect of arginine in preventing the integrity of intestinal function in newborns occurs after feeding piglets milk with a complementary form of arginine, or by metabolic activation of endogenous synthesis of arginine, which both will increase the protein content in skeletal muscle. The study by Kim et al. (2007) found that supplementation with 1% L-arginine to gilts between the thirtieth day of gestation and farrowing increased the viability of piglets by up to 23% and resulted in a 28% increase in weight.

Other experimental studies showed that if supplemented in feed at a dose of 0.2–1% before weaning, it improves the growth and development of piglets (Blavi et al. 2021). At a dosage of 0.4–0.8% in the feed, it supports the DNA synthesis, development of the intestinal mucosa, and the increase in the number of goblet cells in the intestinal mucosa. Moreover, the products of L-arginine (i.e., nitric oxide, creatinine, and polyamines) support the proper function of the intestine, and its regeneration, and prevent intestinal dysfunction (Nuntapaitoon et al. 2018).

However, the economic factors of arginine supplementation must be considered to find the best solution between breeding costs and production profitability. For example, in Brazil, according to Ribeiro et al. (2020), the economic viability of L-arginine supplementation in diets for sows during the lactation phase was counted to be 0.43% of supplementation to be both beneficial and economically possible. According to the same publication, the cost of arginine should not exceed 6.61 times the purchase price of the piglet.

### Tryptophan and leucine

Positive effects on the immune system and antioxidant status were also observed after administration of tryptophan and leucine. For example, when 0.56% tryptophan per kilogram of feed was administered to sows during the last stage of pregnancy until day 7 of lactation, piglet survival increased (Munn et al. 2021). Supplementation of leucine, which is involved in proteosynthesis, is used to increase the amount of protein production in the body (Boutry et al. 2016). Administering 0.8% leucine in the complete feed to sows from day 70 of pregnancy until parturition has been shown to have a positive effect on the weight of newborn piglets, resulting in an increase in their weight (Wang et al. 2018).

In addition, the metabolites of these FAAs support the function of the intestinal barrier and modify the intestinal microflora (Blachier et al. 2013; Munn et al. 2021). It was found that during the inflammatory process in the body, there is increased catabolism of tryptophan by the affected tissue and, therefore, tryptophan is not available for the growth and development of other healthy tissues. For this reason, it is added to the feed in higher amounts than is necessary for the physiological needs of piglets. This supports the possibility of other tissues utilising it for growth purposes (Trevisi et al. 2009).

### Glutamine, glutamate and aspartate

Some FAAs, such as glutamine and glutamate, are considered as conditionally essential, because autogenous synthesis may be insufficient under certain conditions to meet the physiological requirement (Watford 2015; Pluske et al. 2018; Modina et al. 2019). Glutamine is commonly found in the milk and colostrum of sows, in their plasma, and also in amniotic fluid throughout pregnancy. Glutamine can regulate protein degradation, while these degradation products are also substrates for the synthesis of biologically active substances, such as polyamines, glutathione, nucleic acids, hormones, and neurotransmitters, which are essential for animal life, maintaining homeostasis and metabolism processes (Blachier et al. 2013). Lactating and pregnant sows produce more glutamine; indeed, the placenta synthetises and releases a large amount of glutamine into the fetal circulation. Therefore, the administration of glutamine to suckling and weaned piglets is associated with an improvement in overall health and growth acceleration. This is most likely caused by strengthening the function of the intestines and the immune system (Li et al. 2018; Tan et al. 2019).

In a study by Tan et al. (2019), the hypothesis of whether oral administration of glutamate to suckling piglets would affect intestinal morphology was tested. Glutamate was administered in three different concentrations: 0.06 g/kg (low), 0.5 g/kg (medium), and 1 g/kg (high) per day. Jejunum and ileum samples were fixed in formalin for morphometric analysis, while caecal and colon were submitted to volatile fatty acids analysis. The results showed a higher jejunum villus height and crypt depth mainly after the administration of a medium concentration of glutamate at days 7 and 21. Goblet cell number in jejunum increased and, regarding ileum, there was an increase in villus height and a decrease in crypt depth.

Glutamate, glutamine and aspartate provide energy for the development of metabolic processes in the intestines, which use energy in the form of adenosine triphosphate (ATP). After the addition of glutamate, a better development of the intestinal mucosa and an effect on the support of the intestinal barrier (referred to as the tight junction) were observed. Glutamine supplementation prevents intestinal atrophy, supports the activity of intestinal enzymes and improves the growth and development of weaned piglets. Lastly, its catabolism produces a precursor for the formation of polyamines, which are involved in the differentiation, proliferation and repair of intestinal epithelial cells. Glutamine is also a precursor to glutathione, which is an essential antioxidant protecting cells from free oxygen radicals. Positive effects were obtained when feeding 1% glutamine in feed to 21-day-old weaned piglets for 20 days (Hanczakowska and Niwinska 2013).

### Threonine, serine and carnitine

In newborn piglets, an adequate level of threonine is critical to maintain the necessary mucin and IgM production. An increase in threonine content from 8.5 g/kg to 9.0 g/kg beyond the required need led to increased secretion of protective IgM in newborn piglets infected with enterotoxigenic *Escherichia coli* (*E. coli*) (Le Floc’h et al. 2018). However, if the dietary ratio of threonine and lysine between 65% and 70% was changed, weaner pigs were infected by enterotoxigenic *E. coli.* The lack of an impact from complementary threonine can be attributed to mucin-13, expressed in the jejune of pigs, which is not rich in threonine, unlike other mucins that are dominant in the gastrointestinal segment (Le Floc’h et al. 2018).

Serine is classified as a nutritionally non-essential amino acid and is related to both the main metabolism and the route of its synthesis from glucose, the metabolism of which in turn is influenced by the requirement of serine. Its deficiency can result in impaired glycine synthesis, which will lead to a nutritional imbalance of other amino acids (Ji et al. 2020; Blavi et al. 2021). Serine is reported to be the main amino acid needed for the synthesis of a regenerative protein that targets gram-positive bacteria and is abundantly produced in the small intestine of pigs (Blachier et al. 2013; Le Floc’h et al. 2018).

Another of the FAAs group, L-carnitine, supports the development of the placenta, and causes a higher postnatal weight in piglets due to greater muscle development and an increased total number of muscle fibres (Ramanau et al. 2005). It also causes a higher amount of insulin growth factor (IGF), which stimulates and regulates muscle growth and development (Musser et al. 1999). The best results were obtained after a daily administration of 125 mg of L-carnitine to pregnant sows from day 5 to day 112 of pregnancy when an increase in the body weight of newborn piglets was detected (Rooney et al. 2020).

Regards IGF, its levels are also beneficial for the sows’ fertility and piglets’ survival and growth; however, the most promising is its effects on the gut mucosa, where it stimulates growth and repairment and coping with inflammatory changes. Generally, the intestinal mucosa is a critical barrier between the internal and external environment, its impairment lead to increased permeability of the intestine and various health issues (Le Floc’h et al. 2018). Thus, some studies focused their research on the FAAs administration might improve the IGF ratio that can lead to the promotion of growth and development of the intestine. For example, the study by Habidi et al. (2022) proved that a mixture of valine and isoleucine improved gut morphology (i.e., increase in duodenal villus width and crypt depth; ileal villus height and villus width) and potentiated the mRNA expression of IGF in the liver. Similarly, a 1% administration of L-arginine to weaning piglets potentiated the increase in height of the villus in the duodenum, jejunum, and ileum and the overall weight of the intestine increased as well (Yao et al. 2011). However, the resulting effect on intestinal morphology apparently depends on the applied dose of FAAs in diet, since, for example in the Le Floc’h et al. (2018) study, a low 0.3% dose of a mixture of various FAAs did not cause any effect on the IGF plasma level. Even though there were no changes in the level of IGF, the postweaning diarrhoea was milder; thus, the supplementation of FAAs in this study had a demonstrably positive effect on the functioning and balance of the intestinal system.

## THE USE OF FAAs IN WEANING PIGLETS

The FAAs are mainly used in the weaning phase. Weaning is a significant period in the life of piglets as the nutritional supplies differ and piglets must adapt to a variety of challenges arising from an abrupt change from a milk-based to a cerea L-based diet and their digestive system must adapt to the newly established functional aspects (Pluske et al. 2018; Mou et al. 2019; Wessels et al. 2021). The post-weaning period is associated with the occurrence of weaning stress, which can lead to a compromised immune system, and an increased susceptibility to diarrheal diseases, insufficient feed intake, an increase in gastric pH and changes in the composition of intestinal microflora (Pluske et al. 2018). It leads to alterations in the intestinal barrier, a reduction in nutrient absorption and a higher susceptibility to intestinal diseases (Modina et al. 2019). This period of life thus may result in higher morbidity and mortality of piglets due to the development of intestinal disorders associated with diarrhoea, intestinal inflammation, and poor growth of piglets that is mainly related to insufficient nutrition (Mou et al. 2019; Prates et al. 2021). Common morphological changes in the intestine are villous atrophy, crypt hyperplasia, production of lower numbers of goblet cells that are responsible for mucus production, and overall dysbiosis (Wessels et al. 2021). Proper development of the intestine is crucial for proper digestion and immune system function.

The importance to define the ideal protein content in the diet fed during the weaning period is discussed by several authors. Higher protein content in the diet can result in higher production and excretion of nitrogenous metabolites to the environment and the presence of characteristic odour. A low-protein diet can be insufficient in the FAAs and lead to malnutrition and decreased growth (Mou et al. 2019). However, if the low-protein diets are supplemented with well-balanced FAAs, farmers can benefit economically from the health improvement of pigs (Martinez-Aispuro et al. 2022).

For these reasons, in this critical period, it is very important to use growth promoters in piglets, which are able to suppress these problems and thus improve their overall health. We divide them into antibiotic and non-antibiotic growth promoters (Vondruskova et al. 2010). Historically, the use of antibiotics as growth promoters was a common way of support of piglet health. However, the administration of antibiotic growth promoters has been prohibited in all European Union countries since 2006 due to the increasing antibiotic resistance of various types of bacteria and any other possible contamination of the food chain with antibiotic residues. Therefore, they started looking for alternative ways that would replace the use of antibiotic preparations, and at the same time have the same effectiveness and function. Various studies are focused on FAAs supplementation as a promising way to support intestinal development and transmission during the post-weaning period (Beaumont et al. 2022). In fact, the low concentration of FAAs in plasma is associated with the occurrence of runt pigs (He et al. 2016a). Multiple experiments were conducted to support this hypothesis.

### Complementary function of FAAs as a protective to the overall health during weaning

The experiment in the study by Wessels et al. (2021) was based on the hypothesis that FAAs act complementarily and their mixtures can maintain gut balance even at slightly elevated concentrations; however, if not only one but multiple FAAs are elevated. They tested two diets with different mutual ratios of FAAs at a 0.3% concentration above an as-fed basis, specifically arginine, leucine, valine, isoleucine and cystine; in the second diet, tryptophan was added. The methods of evaluation were plasma IGF analysis using an ELISA kit, qPCR analysis of genes related to the barrier function in jejunal tissue, and histological analysis of duodenum, jejunum, and ileum. The results showed an increased crypt depth in the duodenum after the application of both diets and reduced the frequency of diarrhoea and mucin 2 gene expression in the jejunum when the diet with the higher amount of tryptophan was given. Otherwise, the monitored factors were not changed. The conclusion of this study is that 0.3% FAAs supplementation in diet can reduce post-weaning diarrhoea without alteration of growth performance.

In their experiment, Rodrigues et al. (2021) challenged weaned pigs with *Salmonella* Typhimurium verifying the well-known hypothesis, that when the immune system is compromised, the nutrients are relocated to support proper immune system function rather than growth. These authors tested whether the interaction of dietary proteins (at both low and high doses) and FAAs (threonine, methionine, and tryptophan at 120% of requirements) is mutually dependent, and affects growth performance and immune response. The hypothesis was that *Salmonella*-challenged pigs and those fed a high protein diet would have lower performance and poor immune status. Methods applied to validate this hypothesis included the cultivation of rectal swabs and tissue homogenates (mesenteric lymph nodes and spleen), analysis of myeloperoxidase activity as a proinflammatory marker in faecal and digesta samples, growth performance markers (daily gain, daily feed intake), rectal temperature and faecal score, and plasma analysis. In this study, Rodrigues et al. (2021) concluded that FAAs can enhance the growth performance and immunity of pigs regardless of the protein content.

Prates et al. (2021) tested whether the mixture of lipopolysaccharides (LPS) of selected FAAs challenged piglets. The FAAs mixture included arginine, BCAA, and cysteine at a 0.3% concentration above an as-fed basis. The methods of evaluation included gut morphology viewed under a microscope, determination of inflammatory, immune and hormonal markers (i.e., tumour necrosis factor alpha, IGF, immunoglobulins, haptoglobin, cortisol), and proteomic analysis of serum. The FAAs supplementation exerted slight effects on gut morphology because the villus height and crypt depth increased and cortisol decreased.

In addition, the IgG levels increased as the IgM decreased, suggesting the lymphocyte subtypes modification. The IGF increased in response to the FAAs diet; IGF is important for protein synthesis and is stimulated by arginine, as previously reported. Proteomics did not reveal any important modifications. Conclusively, this study suggested that FAAs supplementation can downregulate the inflammatory pathway.

Beaumont et al. (2022) tested a combination of FAAs (arginine, leucine, valine, isoleucine, and cysteine in 0.1% dose) with polyphenols originating from grapes that are known for antioxidant and anti-inflammatory effects in the intestine. The hypothesis was that this combination can facilitate the weaning transition as both these substances positively affect intestinal barrier function and microbiota composition. The methods applied for its evaluation were 16 rRNA gene sequencing and metabolomics of intestinal content, culturing, and gene expression analysis of organoids from intestinal tissue. The combination of FAAs and polyphenols improved the growth and feed efficiency due to an increase in *Lactobacillaceae* and a reduction in the Proteobacteria phylum, upregulated expression of genes responsible for differentiation of epithelia and downregulated expression of innate immunity gene markers.

Li et al. (2018) tested the effect of glutamate and aspartate, in particular in a 21-day lasting experiment. The control group was fed a diet containing 2.9% glutamate and 1.5% aspartate; whereas the experimental group was fed a diet containing 2.6, 3.2, or 3.5% glutamate and 1.3 or 1.7% of aspartate. Growth performance was evaluated by biometric parameters (feed intake and weight), chromatographic determination of amino acids in blood serum, and gene expression analysis in liver tissue samples. The results showed that higher doses of aspartate and glutamate reduce the growth performance in contrast to the low dose of aspartate. Similarly, high doses also reduced amino acids, while a low dose (3.2%) of glutamate, in contrast, increased them. Gene expression of solute carrier family members (7A1, 7A7 and 6A19), which are transporters of neural amino acids, was down-regulated by the highest tested dose of glutamate suggesting the regulation of toxic effects for cells caused by this high dose. In conclusion, the authors claimed that lower doses of glutamate and aspartate are more beneficial for growth performance and nutritional requirements.

### The use of FAAs in the regulation of gut barrier integrity and in maintaining the intestinal health of piglets after weaning

For piglets, the supply of essential nutrients is crucial for optimal growth, intestine development and digestive tract function to prevent gut malabsorption, lower body performance, diarrhoea, immunity disorders, and even death (Mou et al. 2019). A lack of sufficient nutrition can result in intestinal villi atrophy leading to improper functioning of the digestive tract. After weaning, the protein synthesis in the intestine increases and amino acid metabolism alters due to the need to support the tissue development and adaptation during this period (Tang et al. 1999; Le Floc’h et al. 2009; Chalvon-Demersay et al. 2021).

Diarrheal diseases of piglets after weaning can be influenced by dietary measures. It is most important to feed a diet with lower protein content in the post-weaning period, supplement essential amino acids, give probiotics and ensure a sufficient amount of quality water. Furthermore, using feed with a combined content of digestible and indigestible fibre positively affects the composition of the intestinal microflora, improves the digestibility of nutrients and supports the function of the intestinal barrier. Some authors, therefore, recommend using easily digestible and absorbable feed with sufficient amounts of quality nutrients and a low anti-nutritional effect in the post-weaning period. It is described that the use of crude protein in feed with a total content higher than 18% leads to its reduced absorbability by the stomach and small intestine (Cemin 2022). It accumulates in the large intestine, where it serves as a substrate for the growth and reproduction of pathogens, mainly pathogenic strains of *E. coli*. A great deal of attention is also paid to the use of feeds with buffering activity, which affect the ability of the stomach to digest proteins by changing the pH and can influence the growth and reproduction of pathogenic bacteria. Substances that show this buffering activity include, for example, milk products and amino acids. Organic acids are used as additives for an even more significant change in stomach pH.

It was found that in piglets challenged with enterotoxigenic *E. coli,* there was an increase in tryptophan requirement (Trevisi et al. 2009). Also, the endogenous production of arginine is decreased in enterocytes at weaning, which could lead to a shortage of arginine (Wu and Knabe 1995).

#### PRACTICAL USE OF GLUTAMINE IN WEANED PIGLETS

There are several mechanisms by which glutamine and glutamate can contribute to healthy gut development in weaned piglets (Xiong et al. 2019). Both of them are important sources of energy for intestinal epithelial cell proliferation and integrity repair (Hanczakowska and Niwinska 2013; He et al. 2016b) and both serve as oxidative substrates for intestinal epithelial cells and are important sources of carbon atoms for gluconeogenesis (Modina et al. 2019). In the intestine, glutamine may increase secretory IgA production by regulating the intestinal microbiota and/or T-cell-dependent and T-cell-independent pathways (Wu et al. 2016). Glutamine metabolism also provides ATP to support intestinal ion transport, cell growth, and migration (Wu 1998). Catabolism of glutamine provides precursors for the synthesis of polyamine, which is important for the proliferation, differentiation, and repair of intestinal epithelial cells (Lux et al. 1980). Furthermore, glutamine is a precursor for the synthesis of purine and pyrimidine nucleotides, which are essential for DNA synthesis and the proliferation of cells (Wu 1998). Moreover, glutamine is also a precursor for the synthesis of glutathione, an important antioxidant against free radicals (Wu et al. 2004a). The beneficial effects of glutamine on the intestinal tract of weaned piglets were the subject of some experimental studies.

Worth mentioning are the works of American authors comparing the effectiveness of glutamine supplementation with the use of antibiotics in feed for weaned piglets. Their research showed that supplementing 0.2% L-glutamine in the diet for newly weaned and transported piglets either improved (Johnson and Lay 2017) or resulted in similar (Duttlinger et al. 2019; Duttlinger et al. 2020) growth performance compared with a combination of antibiotics (chlortetracycline and tiamulin) used in swine nursery systems following weaning and transport (Puls et al. 2019). Most recently, Duttlinger et al. (2021) found that a 0.2% L-glutamine supplementation tended to improve some morphological markers of intestinal health similarly to antibiotics (chlortetracycline and tiamulin) in piglets following weaning and transport.

According to Wu et al. (1996), dietary glutamine supplementation (1.0%) prevented jejunal atrophy during the first week postweaning and increased the gain : feed ratio by 25% during the second week postweaning. In another study, performed by Hsu et al. (2010), the results showed that the dietary supplementation of 1% or 2% glutamine could be beneficial to small intestinal villous morphology and xylose absorptive capacity of weaned piglets. The villous height in the duodenum and jejunum of the glutamine-exposed groups tended to be higher than that of the control group. The average daily gain increased from approximately 21% to 28% by glutamine supplement compared to the control.

Glutamine supplementation was also useful in reducing the severity of *E. coli* infection in weaned piglets by altering intestinal barrier function and reducing the mucosal cytokine response (Ewaschuk et al. 2011). The authors used dietary supplementation with 4.4% glutamine in weaned piglets for a period of 2 weeks. The exposure of animals to *E. coli* without glutamine addition in their diet resulted in reduced expression of tight-junction proteins; however, this factor was not influenced in glutamine-supplemented piglets. Intestinal tissue from control piglets without glutamine supplementation responded to *E. coli* with a significant increase in mucosal mRNA-encoded cytokines that are markers of inflammatory processes ((i.e., interleukin-1β, interleukin-6, transforming growth factor-β and interleukin-10). No increase in these cytokines was noted in the group that was supplemented with glutamine.

In addition, FAAs can have positive effects on the maintenance of the development of digestive organs and a high turnover rate of cells in weaned piglets (Sauer et al. 2012). The technique which can be used to quantify the trophic action of the dietary additives on tissues uses stable isotopes and measures the carbon turnover rate (Gannes et al. 1998). Assoni et al. (2017) demonstrated by the isotopes technique that the inclusion of 1% glutamine or 1% glutamic acid in piglet diets accelerated the carbon turnover in the stomach during the post-weaning period. It was found that glutamate has guaranteed the fastest 13C incorporation rate on fundic-stomach region.

#### PRACTICAL USE OF SYNTHETIC FORMS OF GLUTAMINE IN WEANED PIGLETS

Although there are many examples of the positive effect of L-glutamine on the piglet digestive system in the scientific literature, its use in practice may be problematic as it is expensive and has low solubility in water and high instability (Modina et al. 2019). In order to eliminate these undesirable properties, synthetic forms, alanyl-glutamine, and glycyl-glutamine, have been developed. These new synthetic forms are more soluble in water, have greater stability, and have a similar or even greater effectiveness than glutamine alone (Furst et al. 1997).

For instance, Zou et al. (2019) found that feeding an alanyl-glutamine diet had beneficial effects on the growth performance, intestinal development and digestive absorption function in weaned piglets. The dietary alanyl-glutamine supplementation at the doses of 0.15%, 0.30%, and 0.45% increased villous height: crypt depth ratio in duodenum and jejunum. It also increased the activities of maltase and lysozyme in the jejunum mucosa.

Another synthetic form of glutamine is glycyl-glutamine. Jiang et al. (2009) conducted a study in order to determine the effects of dietary glycyl-glutamine on postweaning growth, small intestinal morphology, and immune response of stressed or non-stressed weaned piglets. *E. coli* lipopolysaccharide was used as the stress-inducing agent, and the experimental diet was supplemented with 0.15% of glycyl-glutamine.

The authors found that suppression of growth and immune function in piglets challenged with LPS could be alleviated by dietary glycyl-glutamine supplementation.

#### PRACTICAL USE OF ARGININE IN WEANED PIGLETS

Arginine is another FAA that can be used to improve the intestinal barrier. L-arginine supports DNA synthesis and mitochondrial activity of intestinal epithelial cells. In this way, it supports the development of the intestinal mucosa and its regeneration (Nuntapaitoon et al. 2018). Moreover, arginine is required as a substrate for the formation of nitric oxide, creatine, and polyamines (Modina et al. 2019). In general, polyamines support the proper functioning of the intestines; i.e., promote cell proliferation and migration (Matthews 1993; Nowotarski et al. 2013), which is important in mucosal morphology and function, and they also participate in the prevention of intestinal dysfunctions (Liu et al. 2019; Blavi et al. 2021).

For example, Hernandez et al. (2009) found that supplementing pig diets with L-arginine (6 g/kg) improved the post-weaning performance of pigs; i.e., average daily feed intake and average daily gain were increased. The positive effects of arginine supplementation were reported mainly on the intestinal tract of piglets by influencing intestinal morphology. For example, arginine supplementation in weaning piglets contributed to increased villi height in jejunum and ileum, increased ferric-reducing ability of plasma, and lower occurrence of the oxidised form of glutathione by which arginine regulates oxidative stress (Prates et al. 2021).

Another study by Corl et al. (2008) found that oral supplementation of arginine could facilitate mucosal restitution and villus regrowth via stimulation of intestinal p70 S6 kinase in piglets suffering from rotavirus enteritis. Also, Wu et al. (2010) reported that dietary supplementation with 0.6% L-arginine enhanced intestinal growth and integrity (i.e., increased villus height and crypt depth, and increased goblet cell counts in mucosae), and the whole-body weight gain in postweaning pigs.

The positive effects of arginine supplementation under stress conditions were studied as well. Wu et al. (2015) describe the positive effects of dietary supplementation with 1% L-arginine on weaning piglets fed a deoxynivalenol-contaminated (6 mg/kg) diet. The authors found that jejunal morphology together with amino acid concentrations in the serum, jejunum, and ileum were improved.

Another way arginine can influence the development of the intestinal mucosa positively is through its effect on the microvascular development in the small intestine (Zhan et al. 2008). Arginine serves as a precursor of nitric oxide and proline, which are substances important for proper intestinal vascularization (Wu et al. 2004b). In fact, nitric oxide plays an important role in secretion of vascular endothelial growth factor, maintaining of the endothelial function and stimulating of epithelial cell migration (Wu and Meininger 2000). Proline, in addition, plays a crucial role in angiogenesis and vascular remodelling (Adams and Frank 1980). According to Wu et al. (2007), the systemic administration of L-arginine can be considered as a safe and effective method to enhance the synthesis of nitric oxide and proline in piglets. This was also confirmed by Zhan et al. (2008), who found that dietary 0.7% L-arginine supplementation may be a useful method to improve microvascular development in the small intestine of weaning piglets as it increased intestinal villus height and expression of vascular endothelial growth factor in intestinal submucosa and mucosa.

Another practical option involving arginine is its combination with glutamine (Modina et al. 2019). According to Shan et al. (2012), their combination was found to be more effective in preventing villus atrophy and increasing villus height in the small intestine of weaned piglets than the individual application of arginine or glutamine.

#### PRACTICAL USE OF TRYPTOPHAN IN WEANED PIGLETS

Another FAA that has been shown to have a positive effect on the function of the intestinal mucosal barrier is L-tryptophan. Liang et al. (2018) found that dietary tryptophan application (in 0.2–0.4% concentration) can enhance the intestinal mucosal barrier function by increasing the defensin mRNA expression, increasing the abundance of tight junction proteins, and by augmentation of the concentrations of secretory immunoglobulin A. The same study also concluded that tryptophan supplementation resulted in a change in the composition of the bacterial microflora, i.e. that it increased the abundance of bacteria that can utilise tryptophan (*Lactobacillus* and *Clostridium XI*) in the jejunum.

However, there is competition for tryptophan utilisation between skeletal muscle and the immune system, which is the reason why a higher supplementation of tryptophan is needed after a period of poor health (Kampman-van de Hoek et al. 2016).

Tryptophan supplementation was shown to have antioxidant properties that can alleviate the weaning stress syndrome. In general, weaning stress can lead to oxidative damage to macromolecules, i.e., lipids, proteins, and DNA, which can lead to adverse changes in growth and an increase in sensitivity to diseases. Oxidative stress is the result of an imbalance between the endogenous production of reactive forms of oxygen (ROS) and antioxidants (Qi et al. 2020). Tryptophan acts as an antioxidant itself, but several metabolites of tryptophan act as traps with antioxidant properties as well (Kim et al. 2007). Its antioxidant properties were reported when supplemented in a dose above the recommended requirements, i.e. 3.0 g/kg (Martinez-Montemayor et al. 2008).

## THE USE OF FAAS IN GROWING AND FATTENING PIGS

To the best of our knowledge, there are not much data on the use of the FAAs in finishing pigs and their effects on health in the available literature, except for arginine.

For example, a link between oxidative, heat, or slaughter stress in finishing pigs and meat quality was demonstrated. The stress can result in the oxidation of fat and protein and generate malondialdehyde and carbonyl protein complexes that can affect the quality of pork (Lefaucheur et al. 1991; Fang et al. 2002). It was found that arginine can have a positive effect on the meat quality of pigs by reducing oxidative stress; in fact, the dietary use of arginine can increase antioxidant ability, reduce superoxide release, and ameliorate lipid peroxidation (Petrovic et al. 2008).

Another mechanism of the beneficial effect of arginine is that it is metabolized in the organism to nitric oxide, proline, glutamine, and polyamines, as discussed above (Wu and Morris 1998). Physiological levels of these metabolites can attenuate the stress response (Suenaga et al. 2008) and regulate protein synthesis (Yao et al. 2008).

According to Yao et al. (2008), dietary arginine supplementation stimulates protein synthesis and accretion in skeletal muscle of young pigs. An example of a study in which a dietary supplementation of arginine in fattening pigs improved meat quality and attenuated oxidative stress of finishing pigs is a work by Ma et al. (2010). Beginning at 60 kg body weight, pigs were fed a diet supplemented with 1% L-arginine until slaughter (110 kg). These authors found that a dietary supplement of 1% arginine decreased drip loss of pork muscle at 48 h post-mortem, increased intramuscular fat content and enhanced antioxidative capacity and glutathione peroxidase activity in serum. Moreover, 1% arginine increased antioxidative capacity in skeletal muscle.

The practical application of these findings in fattening pigs was implemented in a study by Tan et al. (2009). They found that a 60-day period of supplementation with dietary 1.0% L-arginine increased muscle gain and reduced adiposity in growing-finishing pigs. Compared with the control, arginine supplementation increased body weight gain by 6.5% and carcass skeletal-muscle content by 5.5%. At the same time, it decreased carcass fat content by 11%.

Similar effects were demonstrated in the study by Wu et al. (2012) who found that the lean meat percentage of pigs supplemented with 1% arginine increased by 15.0%, and the fat rate decreased by 34.6%. These effects could be attributed to the fact that arginine plays an important role in regulating the metabolism of energy substrates, and, therefore, nutrient partitioning in mammals (Jobgen et al. 2006).

## CONCLUSIONS

Based on the given data, it is evident that dietary supplementation of functional amino acids has a positive effect on individual categories of pigs. Dietary interventions can be used to support placental growth and proper function, thereby reducing the risk of early embryonic death during pregnancy. Supplementing sows’ diets with functional amino acids during pregnancy has been found to have a positive effect on the development of the placenta, increase fertility, and foetal growth.

Nutritional support for post-weaning intestinal development is a significant part of piglet care. Functional amino acids play a vital role in intestinal growth and development by affecting intestinal morphology, digestive and absorptive functions, and barrier functions. Additionally, dietary supplementation of functional amino acids in fattening pigs can enhance meat quality by reducing oxidative stress. The use of functional amino acids in growing-finishing pigs has also been associated with increased muscle gain and reduced adiposity.
